# Inflammatory Myofibroblastic Tumour: Report of a Rare Form with Exclusive Pleural Involvement

**DOI:** 10.1155/2014/621941

**Published:** 2014-11-26

**Authors:** Gustavo Nobre de Jesus, Sara Lemos Rocha, João Madeira Lopes, João Meneses Santos, Pedro Soares Oliveira, Rui M. M. Victorino

**Affiliations:** ^1^Medicina 2, Hospital de Santa Maria/CHLN, Faculdade de Medicina da Universidade de Lisboa, Avenida Egaz Moniz, 1649-035 Lisboa, Portugal; ^2^Departamento de Anatomia Patológica, Hospital da Luz, Avenida Lusiada, 1500-650 Lisboa, Portugal

## Abstract

Inflammatory myofibroblastic tumour (IMT) is a rare scleroinflammatory lesion, characterized by a myofibroblastic proliferation with inflammatory infiltrates, with many possible locations and diagnosis based on immunohistochemistry. Pleural IMT is uncommon and is usually an extension of a pulmonary involvement. We report on a 28-year-old woman with a new form of this rare entity, characterized by exclusive pleural involvement.

## 1. Introduction

The scleroinflammatory diseases have a wide range of aetiologies and their differential diagnosis is often complex [1]. Inflammatory myofibroblastic tumour (IMT) consists of a myofibroblastic proliferation with variable infiltration of inflammatory cells that may rarely present calcifications [2]. Chromossomic clonal anomalies, histological transformation, and metastasis have been described in case reports, and a recurrence rate as high as 25% has been observed [3]. We report a form of IMT with exclusive pleural involvement that illustrates the complex differential diagnosis of this entity [4, 5].

## 2. Case Report

A 28-year-old female patient presented with a 3-month history of continuous right posterior thoracalgia, with limited response to analgesics. Physical examination showed a pleural rub but was otherwise unremarkable. Laboratory examinations revealed thrombocytosis (511000/mm^3^), erythrocyte sedimentation rate (ESR) of 79 mm, and C-reactive protein (CRP) of 9.24 mg/dL, with normal hepatic and renal function, as well as the remainder of blood count.

**Figure 1:**
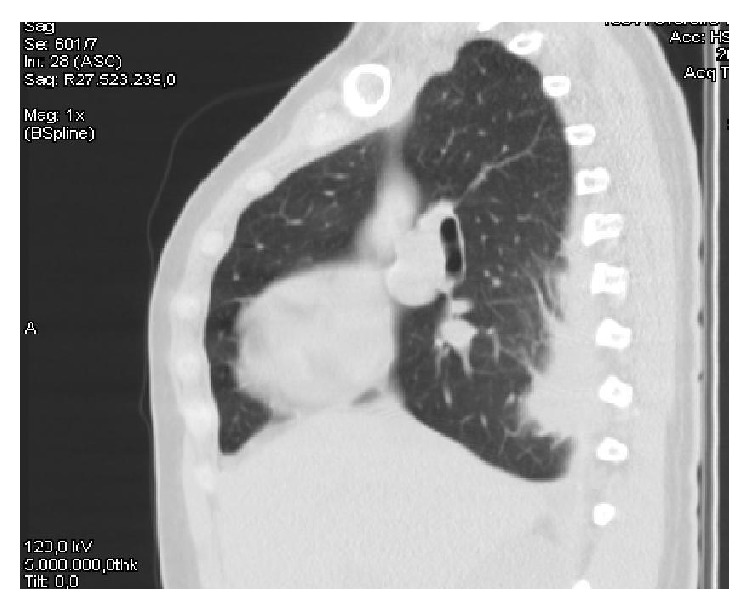
Chest CT with evidence of pleural right lesion.

Abdominal ultrasound and initial chest X-ray were normal. Thoracic CT (Figure 1) showed right posterior pleural thickening, pleural effusion, and passive atelectasis. Further investigation revealed negative IGRA (*Interferon-Gamma Release Assay*) in peripheral blood, as well as sputum and blood cultures. HIV antibodies were negative and no autoantibodies (ANA, ANCA, and anti-DS-DNA) were detected. IgG subclasses determination was normal, with special reference of an IgG4 near the lower limit of normality (6.0 mg/dL).

**Figure 2:**
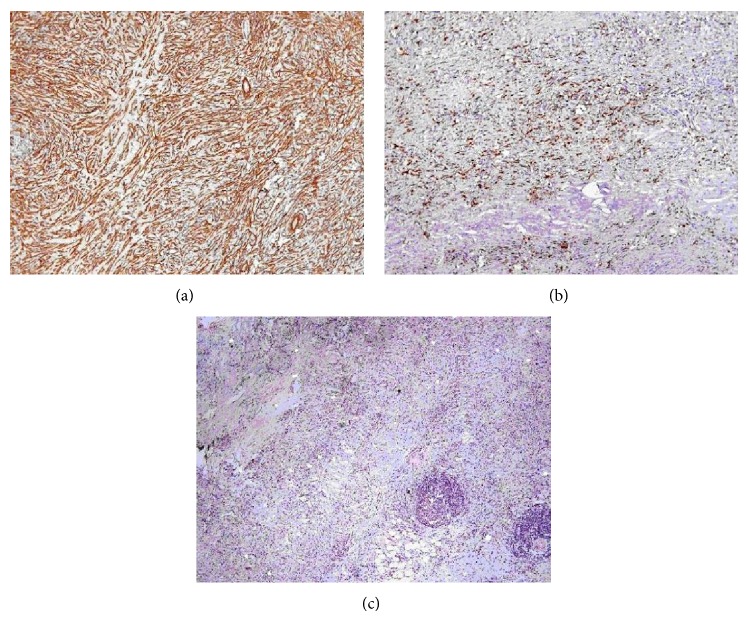
Immunohistochemistry featuring IMT characteristics: diffusely positive for actin, locally positive for FXIIIa, and negative for CD34.

Cultural analysis of CT-guided thoracocentesis was negative, including screening for* Legionella*,* Mycobacterium tuberculosis*, and fungus. Cytology and histology of pleural biopsy revealed nonspecific inflammatory cells and were negative for neoplastic cells. The patient was submitted to surgical removal of the entire pleural mass, which measured 3 × 9 cm. Histopathologic examination revealed an inflammatory hypocellular sclerosing process with disperse lymphoid aggregates. There were no signs of granulomas, calcifications, or neoplastic cells. Immunohistochemistry showed strong focal positivity for vimentin and nonspecific actin, focal positivity for FXIIIa, and negativity for ALK (*anaplastic lymphoma kinase*), CD34, and calretinin (Figure 2). A diagnosis of IMT was established based on the correlation between the morphological and immunocytochemistry findings. Six months after surgery, the patient was asymptomatic, with no evidence of relapse.

## 3. Discussion

IMT is a rare entity, of unknown aetiology, that accounts for less than 1% of all pulmonary tumours [2]. The lung is a commonly affected organ although nonpulmonary locations are well recognized. Pleural involvement has been described but occurs as extension of the pulmonary IMT. One case of possible exclusive pleural involvement has been recently described [5]. But, in contrast to our case, where the mass is strictly pleural, in Loeffler-Ragg's report there was a mediastinal mass with extension to the pleura.

In our case, infectious, neoplastic, and autoimmune aetiologies were initially excluded, as well as IgG4-related disease. Interestingly, calcifying fibrous pseudotumour (CFPT) was a diagnosis initially considered but immunohistochemistry established the final diagnosis of IMT. The differential diagnosis between those two entities was particularly difficult since some clinical and histological characteristics were consistent with CFPT, namely, the absence of systemic symptoms, the unique pleural involvement, and the histological advanced stage of sclerosis. However, the absence of calcifications and the immunohistochemistry confirmed the diagnosis of IMT, although it is noteworthy that a relationship between these two entities has been suggested in previous studies [6].

Fetsh and other authors [4, 6] previously proposed that CFPT could represent a sclerosed end-stage of IMT, as a “burned-out” lesion, similar to other pseudotumours. In fact, both can histologically present with different degrees of calcifications. A case has been reported of a patient with multiple masses containing histological features of both entities and Sigel described a CFPT with focal ALK expression [1, 6–8]. Nevertheless, it is now recognized that there are clear immunohistochemistry differences between CFPT and IMT and a definite relationship has not been established.

The etiopathogenesis of IMT still remains controversial, as illustrated by the frequent changes in nomenclature, the variety of clinical forms, and the diversity of pathological explanations. Patients may present with symptoms such as fever or weight loss, pain, or malaise, although around 70% may be asymptomatic [1]. In the past 10 years, several approaches have been made to investigate the pathogenesis of IMT. Cellular atypia, DNA aneuploidy, and signs of malignancy transformation have been described [3]. Although 30 to 40% are ALK positive and this subgroup has a worse prognosis, a clear relationship with the development of lymphomas has not been confirmed [9, 10]. An infectious-reactive entity has also been proposed (from Epstein-Barr virus to Gram + bacteria), since microorganisms have been identified in some case reports, but again conclusive evidence is still missing [6, 11, 12]. This wide range of clinicohistological forms may suggest that IMT is a spectrum of many entities, including several inflammatory or reactive tumour-like lesions [1, 7, 10].

IMT is considered to be a neoplasm of intermediate biologic potential, which can recur and infrequently metastasize. Histologically, it is characterized by myofibroblastic spindle cells mixed with a hyalinised stroma that appear among various degrees of inflammation infiltrates. Typical immunohistochemistry is diffusely positive for actin, locally positive for FXIIIa, and negative for CD34 [1, 7, 10]. Surgical removal remains the gold-standard therapy. Immunomodulation has been debated as a therapeutical choice since recurrences have been documented up to 11 years after surgery, but it still lacks definite scientific evidence [13, 14]. Given its rarity, there are no guideline-based orientations for diagnosis. We suggest that the diagnostic approach resembles the neoplastic conditions, and clinical suspicion should lead to prompt specific immunohistochemistry studies, critical for definite diagnosis.

In conclusion, the description of this form of exclusive pleural IMT adds to the previously reported clinical spectrum of this rare and poorly understood entity.
